# Fine mapping of the flavonoid 3’,5’-hydroxylase gene controlling anthocyanin biosynthesis in pepper anthers and stems

**DOI:** 10.3389/fpls.2023.1232755

**Published:** 2023-07-27

**Authors:** Yixin Wang, Zheng Wang, Heshan Du, Bin Chen, Guoyun Wang, Qian Wang, Sansheng Geng, Xiaofen Zhang

**Affiliations:** ^1^ Beijing Key Laboratory of Vegetable Germplasms Improvement, Key Laboratory of Biology and Genetics Improvement of Horticultural Crops (North China), National Engineering Research Center for Vegetables, State Key Laboratory of Vegetable Biobreeding, Beijing Vegetable Research Center, Beijing Academy of Agriculture and Forestry Sciences, Beijing, China; ^2^ Department of Vegetable Science, College of Horticulture, China Agricultural University, Beijing, China

**Keywords:** *Capsicum annuum* L, anthocyanins, delphinidin, *ayw*, F3’5’H, CA11g18550

## Abstract

Pepper (*Capsicum annuum* L) is one of the most important vegetables grown worldwide. Nevertheless, the key structural and regulatory genes involved in anthocyanin accumulation in pepper have not been well understood or fine mapped yet. In this study, F_1_, F_2_, BC_1_P_1_, and BC_1_P_2_ pepper populations were analyzed and these populations were derived from a cross between line 14-Z4, which has yellow anthers and green stems, and line 14-Z5, which has purple anthers and stems. The results showed that the yellow anthers and green stems were determined by a single recessive locus called to as *ayw*. While, using preliminary and fine mapping techniques, *ayw* locus was located between markers aywSNP120 and aywSNP124, with physical distance of 0.2 Mb. The *CA11g18550* gene was identified as promising candidate for the *ayw* locus, as it co-segregated with the yellow anthers and green stems phenotypes. *CA11g18550* encodes a homolog of the F3’5’H (flavonoid 3’,5’-hydroxylase) anthocyanin synthesis structure gene. The missense mutation of *CA11g18550* possibly resulted in a loss-of-function. The expression analysis showed that *CA11g18550* was significantly expressed in the stems, leaves, anthers and petals in 14-Z5, and it’s silencing caused the stems changing from purple to green. This study provides a theoretical basis for using yellow anthers and green stems in pepper breeding and helps to advance the understanding of anthocyanin synthesis.

## 1 Introduction

Pepper is an important vegetable because of its pungency and high nutritional value with industrial applications around the world. In 2020, the total harvested area of pepper was 2.07 million hectares, with a production of 36.14 million tonnes (FAO, https://www.fao.org/home/zh/). Many countries rely on male sterile lines to produce hybrid pepper seeds (Dash et al., 2001).

Hybrid breeding uses heterosis to produce varieties that possess both high yield and good quality characteristics (Zhang et al., 2016a). Compared to the creation of hybrid seeds by hand emasculation and pollination, the hybrid system based on male sterility reduces the risk of producing false hybrid seeds resulting from self-pollination (Cheema and Dhaliwal, 2005; Atanassova, 2007). In particular, the release of genic male sterility (GMS) lines is much easier than that of cytoplasmic male sterile (CMS) lines and GMS is less susceptible to ambient temperature compared to CMS (Wei et al., 2019). However, 50% fertile plants need to be pulled out in the field during hybrid seed production. Therefore, it is convenient and economical to differentiate male sterile plants by using Marker-assisted breeding (MAS). Identifying and developing morphological markers associated with traits of interest in breeding is a prerequisite for the successful use of Marker-assisted breeding (MAS) (Zhang et al., 2016a). Such as, the trichome density on the main stem of pepper plants could be used as a morphological marker for assessing PepMoV resistance (Kim et al., 2011), yellow leaves in tomato can be used as a morphological marker for resistance to ToMV (Zhang et al., 2016a). The green hypocotyl is another useful morphological marker in the selection of *male sterile 10* (*ms10*) during seedling evaluation (Zhang et al., 2016a).

Anthocyanins are natural water-soluble pigments that widely exist in plants and are closely related with the color of plant tissues. In plants, there are six widely distributed anthocyanidins, namely cyanidin, delphinidin, malvidin, pelargonidin, peonidin, and petunidin (Zhao et al., 2014). The process of anthocyanin synthesis has been characterized in detail and has been found to be derived from branches of the flavonoid pathway (Petroni and Tonelli, 2011). Two subgroups of related structural genes are involved in the anthocyanin synthesis pathway: early biosynthesis genes (EBGs) and late biosynthesis gene (LBGs) (Petroni and Tonelli, 2011). EBGs, including chalcone synthase (CHS), chalcone isomerase (CHI), and flavanone 3-hydroxylase (F3H), were involved in the synthesis of all downstream flavonoids (Liu et al., 2018). LBGs, flavonoid 3’-hydroxylase (F3’H), flavonoid 3’,5’-hydroxylase (F3’5’H), dihydroflavonol 4-redudase (DFR), anthocyanidin synthase (ANS), and flavonoid 3-O-glucosyltransferase (UFGT) are required for the biosynthesis of anthocyanins (Liu et al., 2018). Dihydrokaempferol could be hydroxylated by F3’H or F3’5’H into dihydroquercitin or dihydromyricetin, respectively. Dihydromyricetin is the precursor of delphinidin, petunidin, and malvidin (Boase et al., 2010). The mechanism of anthocyanin regulation has been extensively studied. Three transcription factors, WD40, bHLH, and R2R3-MYB, joined forces to form the MYB-bHLH-WD40 complex (MBW), which interacts with the promoter of the anthocyanin synthesis gene to control the expression of structural genes and, ultimately, anthocyanin biosynthesis (Li, 2014; Sun et al., 2022).

Currently, pepper fruit color is the primary focus of study on the anthocyanin production of peppers. The *A* locus controlling anthocyanin accumulation in pepper was mapped on chromosome 10, encoding *MYB* transcription factor, which was a homologous gene of *petunia anthocyanin2* (*an2*) (Borovsky et al., 2004). Another gene, anthocyanidin 3-O-glucosyltransferase, was fine-mapped on chromosome 10 and controls anthocyanin synthesis in pepper fruit (Liu et al., 2020). Eight recessive genes (al-1 to al-8) were found to control yellow anthers and green stems, while they have not been cloned yet (Wang and Bosland, 2006). Potato purple pigment generation locus *P* was necessary for blue/purple anthocyanin production, which was localized to chromosomes 11 and codes for F3’5’H (Jung et al., 2005). Several genes that control controlling anthocyanin synthesis have been found in tomato. *Anthocyanin free* (*af*), *anthocyanin reduced* (*are*), *anthocyanin without* (*aw*), *hoffman’ s Anthocyaninless* (*ah*), and *anthocyanin fruit* (*aft*) were mapped by map-based cloning, encoding CHI, F3H, DFR, bHLH, and R2R2-MYB, respectively (Goldsbrough et al., 1994; Kang et al., 2014; Maloney et al., 2014; Qiu et al., 2016; Colanero et al., 2019).

In this study, a pepper line 14-Z4, which has yellow anthers and green stems, was selected from over 1000 pepper accessions. It was then crossed with the pepper line 14-Z5, which has purple anthers and stems, to obtain P_1_, P_2_, F_1_, F_2_, BC_1_P_1_ and BC_1_P_2_ generations for studying the inheritance of anthers and stems color in pepper. After fine mapping, the gene that controls yellow color of anthers and green color of stems was mapped between markers aywSNP120 and aywSNP124, with six candidate genes, among them *CA11g18550* encoded F3’5’H. Furthermore, missense mutations were found in the exon area, and the marker aywSNP550 obtained from *CA11g18550* properly co-segregated with the yellow color of anthers and the green color of stems. Therefore, it can be presumed that *CA11g18550* was a strong candidate gene for controlling the yellow anthers and green stems color.

## 2 Materials and methods

### 2.1 Experimental materials

**Figure 1:**
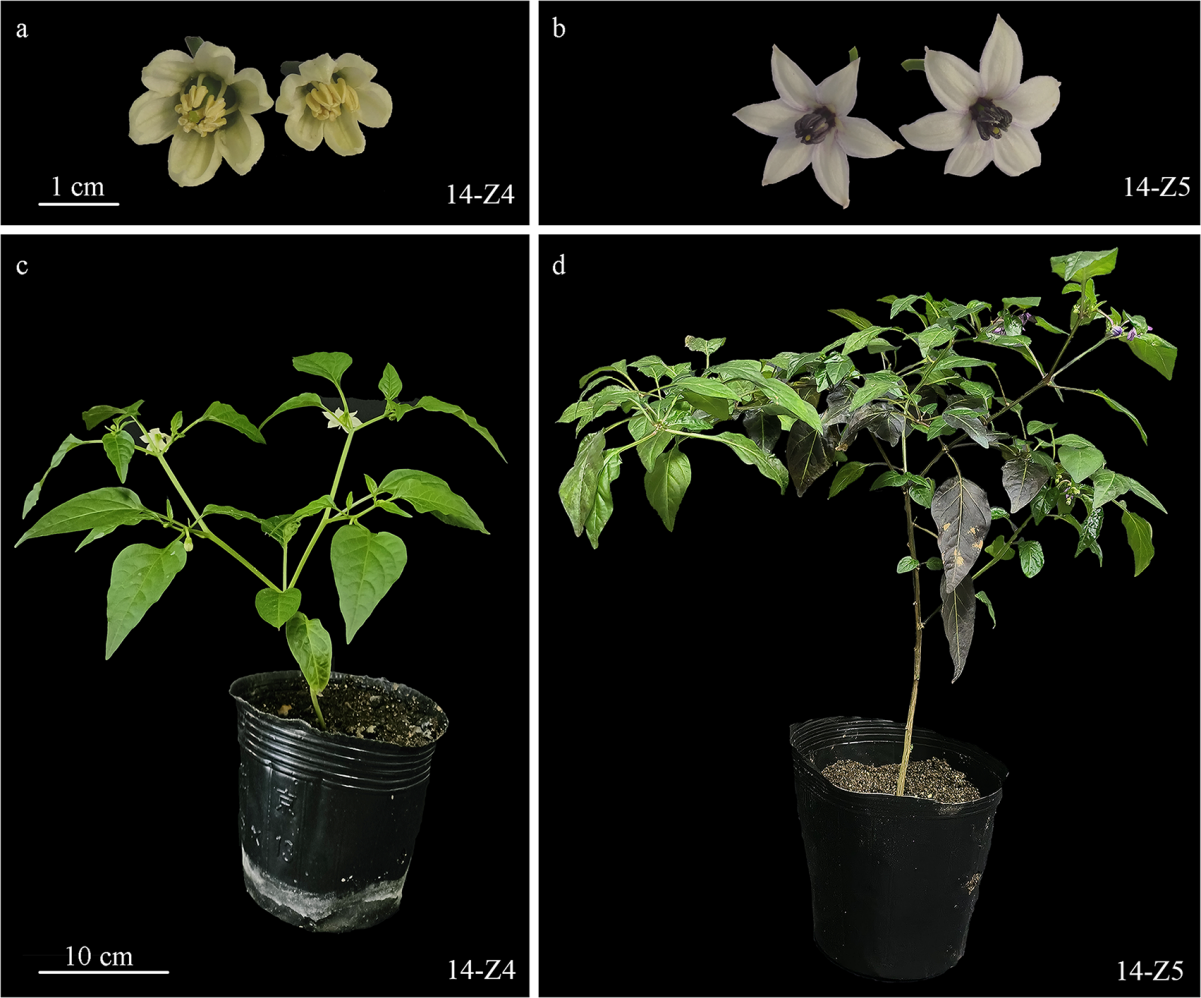
Anthers and plants of 14-Z4 (yellow anthers and green stems) and 14-Z5 (purple anthers and stems). **(A)** Anthers of 14-Z4. **(B)** Anthers of 14-Z5. **(C)** Plant of 14-Z4. **(D)** Plant of 14-Z5.

The experimental materials used in the present study included the *Capsicum annuum* L. inbred parental pepper lines 14-Z4 and 14-Z5 (**Figure 1**) and the F_1_, F_2_, BC_1_P_1_, and BC_1_P_2_ populations derived from reciprocal crosses between the parental lines. Sweet pepper inbred line 14-Z4, which has blocky fruits, yellow anthers and green stems, was provided by the Beijing Vegetable Research Center at the Beijing Academy of Agriculture and Forestry Sciences. The inbred line 14-Z5 was introduced to China from Turin, Italy. This inbred bears horn-shaped fruits in clusters, and has purple anthers and stems. During spring of 2015, the P_1_, P_2_, F_1_, BC_1_P_1_, BC_1_P_2_, and F_2_ generations included 25, 26, 30, 126, 130, and 253 individuals, respectively. During the fall of 2015, the P_1_, P_2_, F_1_, BC_1_P_1_, BC_1_P_2_, and F_2_ generations included 21, 25, 24, 84, 86, and 244 individuals, respectively. During the spring of 2018, the F_2_ generation included 1059 individuals.

A total of 2561 pairs of SSR primers and 185 pairs of InDel primers were developed by our research group using publicly available genomic sequence information for pepper (Zhang et al., 2016b).

### 2.2 Transcriptome library construction and analysis

Anthers were selected on the day of flowering from 14-Z4 and 14-Z5 and immediately frozen in liquid nitrogen. Total RNA was extracted with RNAprep Pure Kit (for plant) (Tiangen, Beijing, China) as described by the manufacturer. The integrity and concentration of RNA were verified using an Agilent 2100 Bioanalyzer (Agilent Technologies, Inc., Santa Clara, CA, USA). Sequencing libraries were constructed following the manufacturer’s instructions of NEBNext ^®^ Ultra ™ RNA Library Prep Kit for Illumina ^®^ (NEB, Ipswich, MA, USA). Sequencing was performed on an Illumina HiSeq 2500 platform (Illumina, USA). Low quality reads were removed. Clean reads were filtered from the raw reads and were mapped to the CM334 genome using Tophat2 software and bowtie2 software (Langmead, 2010; Trapnell et al., 2010; Kim et al., 2013; Kim et al., 2014). Gene expression levels were estimated using FPKM values (fragments per kilobase of exon per million fragments mapped) by RSEM software package (Li and Dewey, 2011). The raw sequencing data generated in this study are available in the NCBI (PRJNA987024).

DESeq and EdgeR were employed and used to evaluate differentially expressed genes (Anders and Huber, 2010). Subsequently, the gene abundance differences between those samples were calculated based on the ratio of the FPKM values. The false discovery rate (FDR) control method was used to identify the threshold of the P-value in multiple tests to calculate the significance of the differences. Here, only genes with an absolute value of log2FC ≥ 1 and FDR < 0.05 were identified as differentially expressed gene (DEGs). DEGs were subjected to Gene Ontology (GO, http://www.geneontology.org/) and Kyoto Encyclopedia of Genes and Genomes (KEGG, https://www.genome.jp/kegg) analysis.

### 2.3 Phenotyping and segregation analysis

Anther colors (yellow or purple) and stem colors (green and purple) were observed in the first flower of each plant at the beginning of flowering. Two different individual researchers recorded anther colors and stem colors in the P_1_, P_2_, F_1_, BC_1_P_1_, BC_1_P_2_, and F_2_ populations at the same time to ensure accurate results. The data were statistically analyzed using Excel 2003, and Chi-squared tests were performed using SAS 8.1 (SAS Institute Inc., Cary, NC, USA).

### 2.4 Chromosomal mapping of the gene controlling anthers and stems coloration

Total genomic DNAs of P_1_, P_2_, F_1_ and F_2_ populations were extracted from young leaves of each plant using the Plant Genomic DNA Kit (Tiangen, Beijing, China). DNAs from 10 individual F_2_ plants with yellow anthers and green stems were pooled and DNAs from 10 individual F_2_ plants with purple anthers and stems were pooled, separately. Before searching for primers to amplify polymorphic products between DNA pools. The reactions for amplifying SSR and InDel markers (10 μL) contained 3 μL of DNA (2.5 ng/μL), 1 μL of each forward and reverse primer (50 ng/μL), and 5 μL of Go Taq^®^ Green Master Mix (Promega, WI, USA). The polymerase chain reaction (PCR) cycling conditions were as follows: pre-denaturation at 94°C for 4 min; 35 cycles of denaturation at 94°C for 15 s, annealing at 55°C for 15 s, and extension at 72°C for 30 s; followed by a final temperature hold at 72°C for 10 min. The amplification products were separated by electrophoresis on 6.0% non-denaturing polyacrylamide gels for 1 h at constant 150 V. The polymorphic primers were used to analyze the genotypes of 253 individuals the F_2_ population at the spring of 2015, and a linkage map was generated using JoinMap 4.0.

### 2.5 Development of co-separation KASPar markers of the *ayw* locus

After mapping the gene to a narrow region, sanger sequencing was used to found the difference sequence between parents in the mapping region, and design KASPar markers based on SNPs. The KASPar platform (LGC Genomics, UK) was used for SNP genotyping individuals from the segregating populations. The conditions for touchdown PCR were as follows: 95°C for 15 min; followed by 10 cycles of denaturation at 94°C for 20 s, and annealing at 61°C (−0.6°C/cycle) for 60 s; and then 26 cycles of denaturation at 94°C for 20 s and annealing at 55°C for 60 s.

### 2.6 Gene expression analysis

Total RNA was extracted using the RNAprep Pure Kit (For Plant) (Tiangen, Beijing, China). First-strand cDNA was synthesized with a PrimeScript™ RT Kit (Takara, Japan). Primers used for the qRT-PCR were designed in Primer5, and primers with product length of 100-300 bp were selected. *UBI-3* (*CA06g03040*) was selected as internal control (Wan et al., 2011) (**Table S1**). The qRT-PCR was performed with a TB Green^®^ Premix Ex Taq™ II (Takara, Japan), on an LightCycler 480 II system. The qRT-PCR was performed with three biological replications and three technical replicates, and relative expression values were calculated using the 2^−ΔΔCt^ method.

### 2.7 Phylogenetic analysis

A phylogenetic tree was constructed with the amino acid sequences of *ayw* and its homologs in other species. All sequences, except *ayw*, were all obtained from the NCBI database. Sequence alignment and tree construction were performed via ClustalW software (version 2.1) and a neighbor-joining tree was constructed using MEGA 7 software (version 7.0.26) with 1000 bootstrap replications.

### 2.8 Virus-induced gene silencing

Virus−induced gene silencing was used to investigate gene function (Liu et al., 2002). Primers were designed using Primer 5 software (forward primer: ACCGAATTCTCTAGATGTTGCTTCTACTCCTAATGCAGCT, and reverse primer: CGTGAGCTCGGTACCTTTTGTGTAATTTTTTCATCCCTCT). The *ayw* fragment (500 bp) was PCR amplified using Primer STAR MAX DNA Polymerase 045A (Takara, Japan) from pepper cDNA. The constructs consisting of pTRV1, pTRV2, pTRV2-PDS, pTRV2-ayw were transformed into *Agrobacterium tumefaciens* GV3101, respectively. The transformed cells were cultured in the Luria–Bertani (LB) medium until the OD600 was 1.0-1.2 and then harvested and re-suspended in the MS buffer (200 μM acetosyringone, 10 mM MES, 10 mM MgCl2, pH = 5.8) to a final OD600 of approximately 1.0. The pTRV1 and the pTRV2, pTRV2-PDS and pTRV2-ayw were mixed in a 1:1 (v/v) ratio, respectively. The mixtures were accomplished using 1 mL syringe without the needle into cotyledons of 3-week-old seedlings. The infiltrated plants were grown in a growth chamber at 22°C, 16 h light/8 h darkness.

## 3 Result

### 3.1 Analysis of the transcriptome sequencing date

To determine the cause of purple deficit in 14-Z4, transcriptome sequencing was performed on anthers from both 14-Z4 and 14-Z5 lines. A total of six samples generated 57769436–71338670 clean reads with an average 41.70% to 42.25% GC content for all libraries. The Q20 and Q30 values exceeded 98.00% and 95.15%, showing the high throughput and quality of the RNA-seq data, respectively (**Table 1**).

**Table 1:** The quality data of transcriptome sequencing.

Sample	Clean Reads	Q20(%)	Q30(%)	GC(%)
14-Z5_1	58,623,164	98.00	95.15	41.95
14-Z5_2	57,769,436	98.10	95.30	42.25
14-Z5_3	65,045,064	98.05	95.25	42.20
14-Z4_1	65,812,624	98.05	95.25	42.00
14-Z4_2	71,338,670	98.05	95.20	41.70
14-Z4_3	62,867,362	98.10	95.30	41.80

### 3.2 Identification of differentially expressed genes

**Figure 2:**
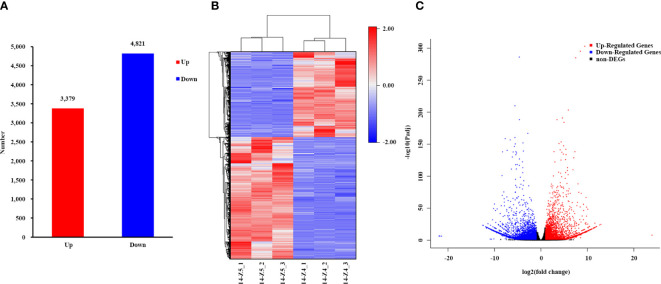
Differential expression analysis in 14-Z4 compared to 14-Z5. **(A)** Then column diagram of the number of upregulated and downregulated genes. The y-axis indicates the number of upregulated and downregulated genes in 14-Z4 vs 14-Z5. **(B)** The cluster heatmap of DEGs in each sample. **(C)** The volcano map of transcriptome. The red dots indicate significantly up-regulated genes, blue dots indicate significantly down-regulated genes, and gray dots indicate genes with no significant difference in expression.

The transcriptome comparison of 14-Z4 vs 14-Z5 identified a total of 8,200 differentially expressed genes (DEGs), including 4,821 DEGs downregulated in 14-Z4 compared to 14-Z5 and 3,379 DEGs upregulated in 14-Z4 compared to 14-Z5 (**Figure 2**).

### 3.3 GO classification statistics and KEGG enrichment analysis

**Figure 3:**
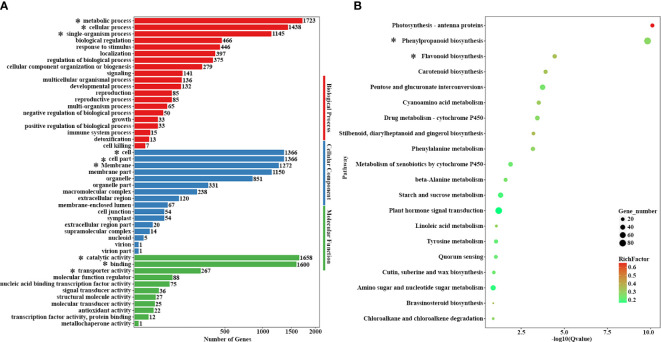
GO classification statistics and KEGG enrichment analysis. **(A)** GO classification analyses of DEGs of 14-Z4 vs 14-Z5. DEGs were classified into three main categories: cellular component, biological process, and molecular function. **(B)** KEGG pathway enrichment analyses of 14-Z4 vs 14-Z5. Important terms and pathways were marked with *.

GO classification statistics and KEGG enrichment analysis were performed to classify the key terms and pathways related to yellow anthers and green stems between 14-Z4 vs 14-Z5. In the 14-Z4 vs 14-Z5 comparisons, 3287 DEGs showed GO annotations. Metabolic process (1723 DEGs), cellular process (1438 DEGs), and single-organizational process (1145 DEGs) were the main terms in the biological process; cell (1366 DEGs), cell part (1366 DEGs), and membrane (1272 DEGs) were the main terms in the cellular component; category activity (1658 DEGs), binding (1600 DEGs), and transporter activity (267 DEGs) were the main terms in the molecular function (**Figure 3A**). To further elaborate the biological interpretation, all DEGs were mapped to the KEGG database (**Figure 3B**). According to KEGG pathway enrichment analysis we obtained 12 significantly enriched pathways, among them photosynthesis-antenna proteins, phenylpropanoid biosynthesis, and flavonoid biosynthesis were three predominant KEGG pathways. Phenylpropanoid biosynthesis was an upstream pathway of anthocyanin synthesis, and anthocyanin synthesis was derived from branches of the flavonoid pathway (**Figure 3B**). Therefore, the color difference between 14-Z4 and 14-Z5 was due to the anthocyanin synthesis.

### 3.4 Genetic analysis of yellow anthers and green stems trait in pepper

The F_1_ plants crossed by “14-Z5” (yellow anthers and green stems) and “14-Z5” (purple anthers and stems), the segregation of purple anthers and stems phenotype. In the F_2_ populations observed during the spring of 2015, a total of 184 plants with purple anthers and stems and 69 plants with yellow anthers and green stems were observed, and the ratio of plants with purple anthers and stems to those with yellow anthers and green stems was consistent with the 3:1 ratio expected for segregation of a single gene, according to a Chi-squared test (**Table 2**). Similarly, during the fall of 2015, a total of 186 plants with purple anthers and stems and 57 plants with yellow anthers and green stems were observed, and the ratio of plants with purple anthers and stems to those with yellow anthers and green stems in the F_2_ was also consistent with the expected ratio of 3:1 according to a Chi-squared test (**Table 2**). In the BC_1_P_1_ population, for both spring and fall 2015, the proportion of plants with yellow anthers and green stems to those with purple anthers and stems was 1:1. In the BC_1_P_2_ population in the spring and fall of 2015, the anthers and stems of all plants were purple (**Table 2**). These findings suggest that yellow anthers and green stems co-segregated, and are controlled by a recessive nuclear gene called *ayw*.

**Table 2:** Segregation ratios of plants with purple anthers and stems or yellow anthers and green stems in populations derived from the 14-Z4×14-Z5 cross.

Populations	Plants tested		Yellow anthers and green stems plants		Purple anthers and stems plants		Expected		X^2^		P-value	
	Spring	Fall	Spring	Fall	Spring	Fall	Spring	Fall	Spring	Fall	Spring	Fall
14-Z4	25	21	25	21	0	0	–	–	–	–	–	–
14-Z5	26	25	0	0	26	25	–	–	–	–	–	–
14-Z4×14-Z5	30	24	0	0	30	24	–	–	–	–	–	–
(14-Z4×14-Z5)×14-Z4	126	84	61	41	65	43	1:1	1:1	0.13	0.05	0.72	0.83
(14-Z4×14-Z5)×14-Z5	130	86	0	0	130	86	–	–	–	–	–	–
F_2_ in 2015	253	244	69	58	184	186	1:3	1:3	0.70	0.20	0.40	0.66
F_2_ in 2018	1059	–	275	–	784	–	1:3	–	0.54	–	0.47	–

### 3.5 Preliminary chromosomal mapping of the pepper *ayw* locus

**Figure 4:**
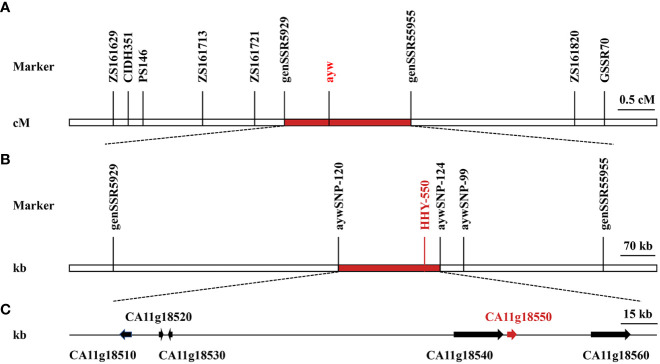
Genetic and physical maps of *ayw* locus and analysis of candidate genes. **(A)** Genetic map of *ayw* locus was generated by 253 F_2_ individuals using joinmap 4.0. **(B)** Physical map of *ayw* locus was further fine mapped using KASPar markers. **(C)** Candidate genes in the fine mapping interval.

To distinguish SSR and InDel alleles, 2561 pairs of primers were used to screen parental lines 14-Z4 and 14-Z5 for polymorphisms, resulting in a selection of 357 pairs of polymorphic primers with a polymorphism rate of 13.00%. The pooled DNAs of plants with yellow anthers and green stems or purple anthers and stems were each analyzed using these 357 pairs of polymorphic primers, from which nine pairs of polymorphic primers (including eight pairs of SSR primers and one pair of InDel primers), with a polymorphism rate of 2.52%, were identified (**Table S2**). These nine pairs of polymorphic primers were then used for marker analysis and to construct a linkage map in F_2_ population comprised of 253 individual plants. A linkage map of 6.6 cM was constructed and the *ayw* locus was mapped on pepper chromosome 11 between marker genSSR5929 and marker genSSR5955, which were 0.4 cM and 1.4 cM from *ayw* locus, respectively (**Figure 4**).

### 3.6 Validation of the marker co-segregating with the pepper *ayw* locus

In the spring of 2018, the F_2_ population of 1059 plants was employed for fine mapping. Following sanger sequencing of PCR results, sequence variations were detected between markers SSR5929 and SSR5955 in the two parental lines, resulting in the identification of three SNPs. Corresponding to these SNPs, 3 KASPar markers were designed and they were polymorphic between the parental lines and F_2_ populations (**Table S3**). Further linkage analysis was conducted on F_2_ populations it was determined that the *ayw* locus was located between marker aywSNP120 and marker aywSNP124, which were physically separated by a distance of 0.2 Mb (**Figure 4**).

**Figure 5:**
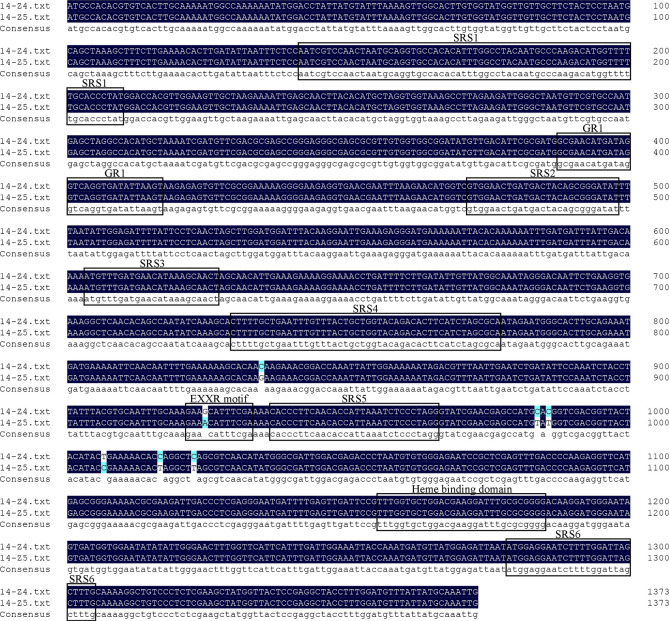
The cDNA sequence alignment of *CA11g18550* between 14-Z4 and 14-Z5.

According to the gene annotation of the CM334 reference genome, a total of 6 putative genes (*CA11g18510*, *CA11g18520*, *CA11g18530*, *CA11g18540*, *CA11g18550*, and *CA11g18560*) were located in the 0.2 Mb region (**Figure 4**). According to the annotation, a gene named *CA11g18550* was annotated as F3’5’H, which was a structural gene in the anthocyanin synthesis pathway (**Table 3**). Sequencing results showed that the sequence of *CA11g18550* was different between 14-Z4 and 14-Z5 (**Figure 5**). Based on the SNP differences of sequencing at positions + 835 bp, KASPar marker aywSNP550 was designed and the marker was identified to co-segregated with phenotype (**Table S3**).

**Table 3:** Annotation of six genes between marker aywSNP120 and aywSNP124.

Gene ID	Start	End	Annotation
CA11g18510	254589739	254593893	Mitochondria isoform 1 [Theobroma cacao]
CA11g18520	254610377	254610637	Aspartyl protease family protein
CA11g18530	254611482	254611718	Orf315 protein
CA11g18540	254730719	254730719	Vacuolar protein sorting vps41, putative
CA11g18550	254751075	254753668	Flavonoid 3’,5’-hydroxylase
CA11g18560	254789195	254797042	Lysosomal Pro-X carboxypeptidase, putative

The sequence alignment of the exon in parental lines revealed nucleotide substitutions in *CA11g18550*. Mutations at positions + 984 bp, + 1017 bp, + 1023 bp were a synonymous mutation, while mutations at positions + 835, + 925, + 986, + 1007 resulted in changes in the 279th, 309th, 329th, 336th amino acid residue, respectively. These substitutions changed the amino acids from glutamic acid (GAA) to glutamine (CAA) to, from threonine (ACA) to alanine (GCA), from methionine (ATG) to threonine (ACG) to, from proline (CCG) to leucine (CTG) (**Figure 5**).

### 3.7 Evolution and expression analysis

**Figure 6:**
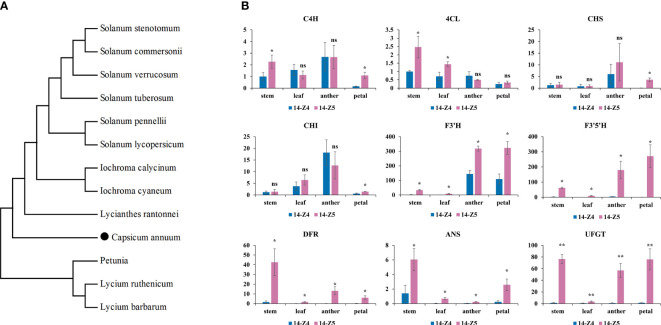
Phylogenetic tree of F3’5’H and transcriptional level of anthocyanin structure gene. **(A)** Phylogenetic tree of F3’5’H proteins in pepper and other species. Accession number for the respective protein sequences were as follows: *Lycium ruthenicum* (AGT57963.1); *Iochroma calycinum* (AIY22750.1); *Lycium barbarum* (AGT57962.1); *Iochroma cyaneum* (AIY22752.1); *Lycianthes rantonnei* (AAG49300.1); *Solanum pennellii* (NP_001361341.1); *Solanum stenotomum* (XP_049380725.1); *Solanum lycopersicum* (NP_001234840.2); *Solanum verrucosum* (XP_049353506.1); *Solanum commersonii* (KAG5577898.1); *Solanum tuberosum* (BAJ09746.1); *Petunia* x *hybrida* (P48419.1). *CA11g18550* was highlighted with black dot. **(B)** Transcriptional level of anthocyanin structure gene in stems, leaves, anthers and petals between 14-Z4 and 14-Z5. The error bars represent the standard deviation of measurements for three replicates in 14-Z4 and 14-Z5. Asterisks indicated a statistically significant difference between varieties (*p < 0.05; **p < 0.01; ns p ≥ 0.05; t-test).

*CA11g18550* encodes for F3’5’H, a structural gene in the anthocyanin synthesis pathway. A BLAST search (NCBI) performed with the coding sequence was performed using the coding sequence and a phylogenetic tree was generated from the protein sequences from the NCBI database (**Figure 6A**). The results showed that *CA11g18550* had a high level of similarity with *Lycianthes rantonnei* and *Petunia* x *hybrida*, with the percent identity of 92.58% and 90.31%, respectively.

To further investigate the expression pattern of *CA11g18550*, stems, leaves, petals and anthers of the two parents were analyzed by qRT-PCR. The results showed that *CA11g18550* was expressed at a significantly higher level (>37 times) in anthers of 14-Z5 than in 14-Z4 (**Figure 6B**). Additionally, the expression of other genes involved in anthocyanin synthesis (*F3’H*, *F3’5’H*, *DFR*, *ANS*, and *UFGT*) in 14-Z5 was significantly more than twice as high as in 14-Z4 in stems, leaves, anthers, and petals. However, the expression of *C4H*, *4CL*, *CHS*, and *CHI* genes did not show any relation to purple color (**Figure 6B**).

### 3.8 VIGS experimental verification

**Figure 7:**
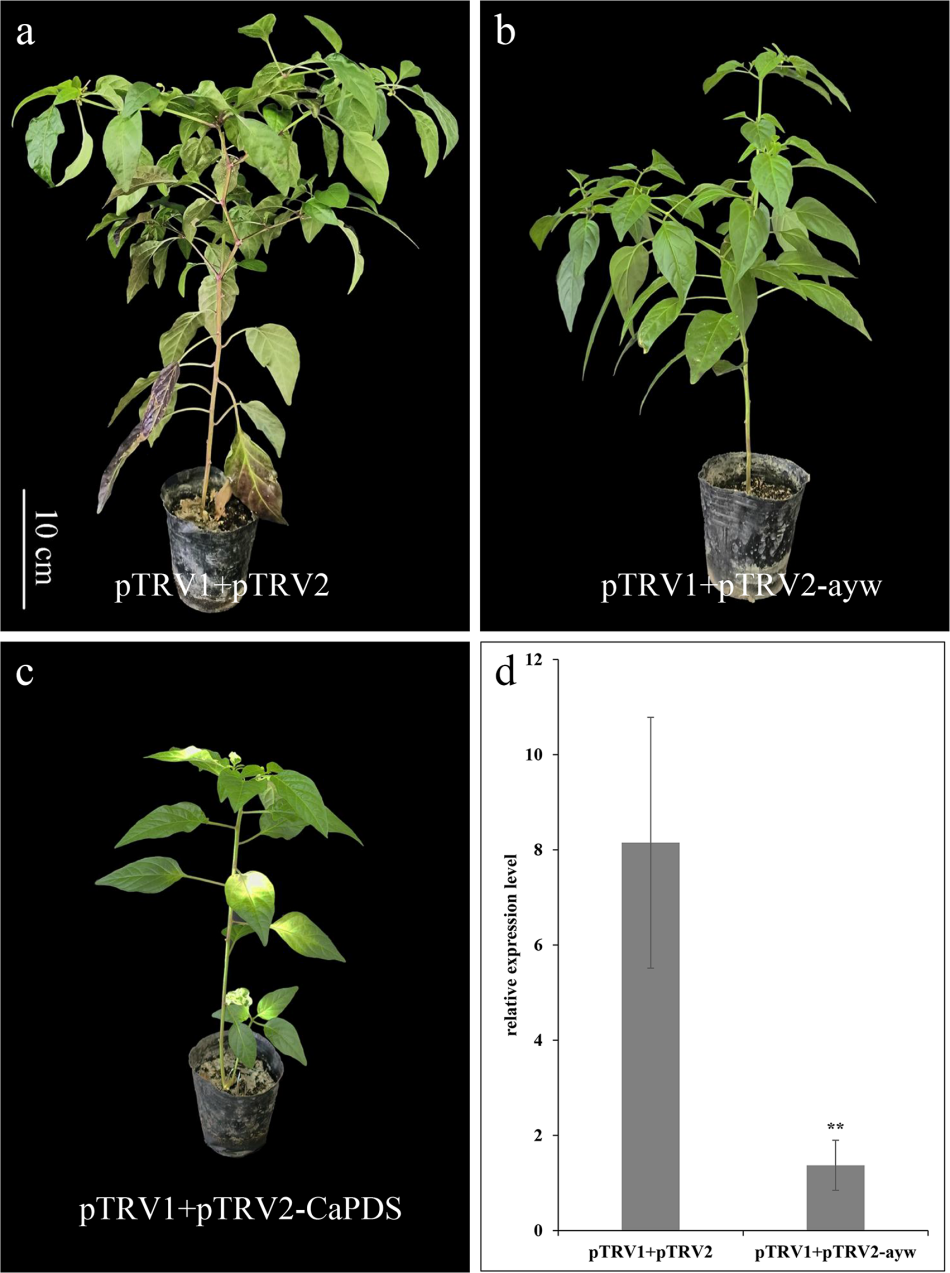
Silencing of *ayw* led to the color of the stem changes from purple to green. **(A)** pTRV1+pTRV2 infiltrated plants (negative control). **(B)** pTRV1+pTRV2-ayw infiltrated plants. **(C)** pTRV1+pTRV2-CaPDS infiltrated plants (positive control). **(D)** The qRT-PCR analysis of *ayw* in pTRV1+pTRV2-ayw infiltrated and control (pTRV1+pTRV2) plants. Asterisks indicated a statistically significant difference between varieties (**p < 0.01; t-test).

Virus induced gene silencing was used to observe whether the *ayw* locus was related to anthocyanin accumulation. The positive control, pTRV1 + pTRV2-CaPDS was employed to confirm the success of gene silencing (**Figures 7A, C**). The expression level of *ayw* in pTRV1 + pTRV2 plants was found to be 5.97 times higher compared to pTRV1 + pTRV2-ayw plants, indicating that VIGS could effectively silence *ayw* (**Figure 7D**). The stem of pTRV1 + pTRV2 plant has obvious purple, while the stem of pTRV1 + pTRV2-ayw plant appeared green (**Figure 7B**), indicating that the down-regulation of *ayw* could reduce the accumulation of anthocyanins in pepper.

## 4 Discussion

Anthocyanin synthesis and regulation have been extensively documented, and some genes affecting fruit color in pepper have been uncovered (Borovsky et al., 2004; Liu et al., 2020). However, the regulation of anthers color and stems color in pepper is still unclear. In this work, a molecular genetic investigation of pepper anthers and stems was carried. The research was conducted in reciprocal cross F_1_, BC_1_P_1_, BC_1_P_2_, and F_2_ populations developed from parental pepper lines 14-Z4, which has yellow anthers and green stems, and 14-Z5, which has purple anthers and stems. According to the data from three seasons, the yellow anthers and green stems were identified to be co-segregated. Through map-based cloning, a recessive gene *ayw*, which encodes F3’5’H, was identified as the major candidate gene influencing the yellow color of the anthers and the green color of the stems after preliminary and fine mapping. Our research provides a useful reference for clarifying the synthesis of anthocyanins in pepper, and also provides important theoretical support for the application of green stem as a seedling marker in pepper GMS breeding.

### 4.1 Delphinidin makes pepper purple

Plant pigments such as betalains, carotenoids, and anthocyanins are responsible for the diverse color found in plants (Grotewold, 2006; Tanaka et al., 2008; Zhao et al., 2022). Red violet betacyanins and the yellow betaxanthins are immonium conjugates of betalamic acid with cyclo-dopa and amino acids or amines, respectively (Strack et al., 2003). Carotenoids mainly provide yellow to orange colors for plants, and anthocyanins provide the majority of the orange, red, purple, and blue colors for plants (Grotewold, 2006). In this study, we analyzed the transcriptomes of anthers from the pepper lines 14-Z4 and 14-Z5. The results showed that flavonoid biosynthesis and phenylpropanoid biosynthesis were significantly enriched. These two KEGG pathways are related to anthocyanin synthesis and anthocyanins were derived from a branch of the flavonoid pathway. The results showed that the difference in color between 14-Z4 and 14-Z5 is caused by the difference in anthocyanin content.

Finally, we were able to map the *ayw* locus between aywSNP120 and aywSNP124 on chromosome 11, which included six candidate genes. Among these genes, one gene named *CA11g18550* attracted our great attention based on annotation. Additionally, a marker aywSNP550, which was derived from *CA11g18550*, perfectly co-segregated with the yellow color of the anthers and green color of the stems. *CA11g18550* encodes F3’5’H, which is member of the cytochromes P450 family. It converts dihydrokaempferol and dihydroquercetin into dihydromyricetin, which is is the precursor of delphinidin (Peng et al., 2019). In carnations, chrysanthemums and other flowers, blue/purple flower colors could not be produced due to the lack of delphinidin. The purple tubers of potatoes and purple flowers and colors of petunias could not be separated from delphinidin. Delphinidin is the main anthocyanin in the flowers, stems and fruits of purple pepper. Studies have shown that delphinidin derivatives are the only anthocyanins present in purple/black pepper fruits. The most common anthocyanin structure in pepper fruit was delphine-3-p-coumarinyl-rutinoside-5-glucoside (Lightbourn et al., 2008; Stommel et al., 2009). Therefore, the synthetic pathway of delphinidin is the key pathway of purple color in pepper. The hydroxylation of F3’5’H at the 5’ - position is a particularly important step, which determines the synthesis of delphinidin in plants (Wang et al., 2014). Only plants containing delphinidin could display blue/purple color (Jung et al., 2005; Sato et al., 2011; Brugliera et al., 2013). Many studies have shown that introducing the F3’5’H could improve flower color and cultivate blue carnations, chrysanthemums and other flowers (Mori et al., 2004; Brugliera et al., 2013; Chandler et al., 2013). When the F3’5’H of pansy was transferred, the pink chrysanthemum lacking F3’5’H was transformed into the transgenic blue chrysanthemum which could produce anthocyanins accumulating delphinidin (Brugliera et al., 2013). When the F3’5’H gene cloned from Vinca major was expressed in transgenic *Petunia hybrida*, some transgenic plants show dramatic color changes from red to dark purple (Mori et al., 2004). In addition, the blue/purple color of other plant organs was also usually due to the accumulation of delphinidin. It has been proven that *P* gene of potato and the F3’5’H gene of tomato were located in the same region in potato and tomato genomes, and F3’5’H gene was co-segregated with tuber purple character in F_1_ subpopulation in potato (Jung et al., 2005). Further transgenic results showed that overexpression of F3’5’H gene could make potato have purple stems and purple tubers (Jung et al., 2005).

In addition, there were four missense mutations in exon of *CA11g18550* between 14-Z4 and 14-Z5, and the KASPar marker aywSNP550 was designed according to the SNP mutation on +835bp of *CA11g18550*, which co-segregated with the color of anthers and stems of the F_2_ population. The expression level of *CA11g18550* in purple anthers and stems was significantly higher than in yellow anthers and green stems. At the same time, silencing *CA11g18550* through VIGS causes purple stems to turn green (**Figure 7B**). Therefore, based on all the evidence above, *CA11g18550* was identified as a strong candidate gene for *ayw* locus in pepper.

### 4.2 Stem color controlled by single gene is a useful phenotypic marker in plant breeding

Previous studies have shown that recessive traits controlled by single genes could be useful for crop breeding (Cheema and Dhaliwal, 2005; Gardner and Panthee, 2010; Saxena et al., 2011; Su et al., 2012; Panthee and Gardner, 2013; Wan et al., 2015; Zhang et al., 2016a). The homozygosity of the inbred parents of F_1_ hybrid seeds has an immediate impact on yield and quality. The use of male sterile lines throughout the hybrid seed production process can help avoid false hybrid seeds produced by self-pollination, increase seed quality, and lower production costs. However, CMS needs three lines of sterile line, maintainer line and restorer line, which difficult to breed and are susceptible to ambient temperature. GMS can avoid the defects of CMS, while needs 50% fertile plants need to be pulled out in the field during hybrid seed production. Morphological markers inherited as a single recessive gene, which linked to these traits could be used to distinguish selfed seedlings from true hybrids, to test the genetic composition of parental lines and hybrid seeds, and to identify sterile males during early developmental stages (Cheema and Dhaliwal, 2005; Gardner and Panthee, 2010; Saxena et al., 2011; Su et al., 2012; Panthee and Gardner, 2013; Wan et al., 2015; Zhang et al., 2016a). For example, in rice, the phenotype of the gry79 mutant was controlled by a single recessive nuclear gene, and its mutant phenotype was useful for discriminating false hybrids (Wan et al., 2015). Zhang et al. (2016a) developed a seedling morphological marker from the single recessive gene *aa* for the MAS of the *male sterile 10* gene in tomato. However, identifying pepper hybrids, as well as male sterile and maintainer lines can be complicated, time consuming, and costly. Anther color of pepper could be used as a marker character to distinguish male sterility. A nuclear male sterile line (MS-12) was associated with 3 morphological markers-taller plant height, erect plant growth habit, and dark purple anthers. Male sterile plants with reduced plant height and moderate growth behaviour could be selected during the early growth stage. However, individual variances in plant height and growth habits that were easily influenced by the environment, this could result in the escape of composite male viable plants with tall height during the initial monitoring in the hybrid seed production field. Thus, the anther color was used for the second monitoring for further identification in the later stage (Dash et al., 2001). Selection of male sterile plants at the flowering stage was expensive and time-consuming and too late. In contrast, selection of male-sterile plants at the seedling stage allows only male sterile plants to be transplanted in hybrid seed production block economizing area and labor (Cheema and Dhaliwal, 2005). Our study showed that the *ayw* locus controls yellow anthers and green stems. The anthocyaninless mutants (al-1 to al-8) also showed that yellow anthers and green stems were controlled by the same recessive genes, even though there was no clear gene location (Wang and Bosland, 2006). Therefore, green stems, a readily identifiable recessive marker characteristic, could be transmitted to pepper male sterile lines, allowing male sterile lines to be distinguished by stem color at the seedling stage and avoiding the removal of 50% fertile lines after field transplanting. As a result, it is possible to distinguish male sterile lines by stem color at the seedling stage and avoid the removal of 50% fertile lines after transplanting in the field.

In summary, in this study identified *CA11g18550* as a strong candidate gene of *ayw* through various methods including mapping, RNA-seq, and gene expression analysis. This provides a useful reference for the future research on anthocyanins, and lays a foundation for using green stem as morphological marker in pepper assisted breeding.

## Data availability statement

The datasets presented in this study can be found in online repositories. The names of the repository/repositories and accession number(s) can be found below: NCBI BioProject, accession PRJNA987024. The names of the repository and accession number can be found in the article.

## Author contributions

XZ, HD and SG contributed to the experimental design. YW and GW performed experiments, analyzed results. QW and BC participated in experimental design and statistical analysis. YW and ZW participated in manuscript writing. All authors read and approved the final manuscript.

## References

[B1] Anders S. Huber W. (2010). Differential expression analysis for sequence count data. Genome Biol. 11 (10), R106. doi: 10.1186/gb-2010-11-10-r106 20979621 PMC3218662

[B2] Atanassova B. (2007). Genic male sterility and its application in tomato (*Lycopersicon esculentum* mill.) hybrid breeding and hybrid seed production. Acta Hortic. 729), 45–51. doi: 10.17660/ActaHortic.2007.729.3

[B3] Boase M. R. Lewis D. H. Davies K. M. Marshall G. B. Patel D. Schwinn K. E.. (2010). Isolation and antisense suppression of *flavonoid 3', 5'-hydroxylase* modifies flower pigments and colour in cyclamen. BMC Plant Biol. 10, 107. doi: 10.1186/1471-2229-10-107 20540805 PMC3095274

[B4] Borovsky Y. Oren-Shamir M. Ovadia R. De Jong W. Paran I. (2004). The *A* locus that controls anthocyanin accumulation in pepper encodes a *MYB* transcription factor homologous to *Anthocyanin2* of Petunia. Theor. Appl. Genet. 109 (1), 23–29. doi: 10.1007/s00122-004-1625-9 14997303

[B5] Brugliera F. Tao G. Q. Tems U. Kalc G. Mouradova E. Price K.. (2013). Violet/blue chrysanthemums–metabolic engineering of the anthocyanin biosynthetic pathway results in novel petal colors. Plant Cell Physiol. 54 (10), 1696–1710. doi: 10.1093/pcp/pct110 23926066

[B6] Chandler S. F. Senior M. Nakamura N. Tsuda S. Tanaka Y. (2013). Expression of flavonoid 3',5'-hydroxylase and acetolactate synthase genes in transgenic carnation: assessing the safety of a nonfood plant. J. Agric. Food Chem. 61 (48), 11711–11720. doi: 10.1021/jf4004384 23646984

[B7] Cheema D. S. Dhaliwal M. S. (2005). Hybrid tomato breeding. J. New Seeds 6 (2-3), 1–14. doi: 10.1300/J153v06n02_01

[B8] Colanero S. Tagliani A. Perata P. Gonzali S. (2019). Alternative splicing in the *anthocyanin fruit* gene encoding an R2R3 MYB transcription factor affects anthocyanin biosynthesis in tomato fruits. Plant Commun. 1 (1), 100006. doi: 10.1016/j.xplc.2019.100006 33404542 PMC7747991

[B9] Dash S. S. Kumar S. Singh J. N. (2001). Cytomorphological characterization of a nuclear male sterile line of chilli pepper (*Capsicum annuum* L.). CYTOLOGIA 66 (4), 365–371. doi: 10.1508/cytologia.66.365

[B10] Gardner R. G. Panthee D. R. (2010). Grape tomato breeding lines: NC 1 grape, NC 2 grape, and NC 3 grape. HortScience 45 (12), 1887–1888. doi: 10.21273/HORTSCI.45.12.1887

[B11] Goldsbrough A. Belzile F. Yoder J. I. (1994). Complementation of the Tomato *anthocyanin without* (*aw*) Mutant Using the Dihydroflavonol 4-Reductase Gene. Plant Physiol. 105 (2), 491–496. doi: 10.1104/pp.105.2.491 12232217 PMC159386

[B12] Grotewold E. (2006). The genetics and biochemistry of floral pigments. Annu. Rev. Plant Biol. 57, 761–780. doi: 10.1146/annurev.arplant.57.032905.105248 16669781

[B13] Jung C. S. Griffiths H. M. De Jong D. M. Cheng S. Bodis M. De Jong W. S. (2005). The potato *P* locus codes for flavonoid 3',5'-hydroxylase. Theor. Appl. Genet. 110 (2), 269–275. doi: 10.1007/s00122-004-1829-z 15565378

[B14] Kang J. H. McRoberts J. Shi F. Moreno J. E. Jones A. D. Howe G. A. (2014). The flavonoid biosynthetic enzyme chalcone isomerase modulates terpenoid production in glandular trichomes of tomato. Plant Physiol. 164 (3), 1161–1174. doi: 10.1104/pp.113.233395 24424324 PMC3938611

[B15] Kim H. J. Han J. H. Kim S. Lee H. R. Shin J. S. Kim J. H.. (2011). Trichome density of main stem is tightly linked to PepMoV resistance in chili pepper (*Capsicum annuum* L.). Theor. Appl. Genet. 122 (6), 1051–1058. doi: 10.1007/s00122-010-1510-7 21184049

[B16] Kim S. Park M. Yeom S. I. Kim Y. M. Lee J. M. Lee H. A.. (2014). Genome sequence of the hot pepper provides insights into the evolution of pungency in *Capsicum* species. Nat. Genet. 46 (3), 270–278. doi: 10.1038/ng.2877 24441736

[B17] Kim D. Pertea G. Trapnell C. Pimentel H. Kelley R. Salzberg S. L. (2013). TopHat2: accurate alignment of transcriptomes in the presence of insertions, deletions and gene fusions. Genome Biol. 14 (4), R36. doi: 10.1186/gb-2013-14-4-r36 23618408 PMC4053844

[B18] Langmead B. (2010). Aligning short sequencing reads with Bowtie. Curr. Protoc. Bioinf. Chapter 11, Unit–11.7. doi: 10.1002/0471250953.bi1107s32 PMC3010897 21154709

[B19] Li S. (2014). Transcriptional control of flavonoid biosynthesis: fine-tuning of the MYB-bHLH-WD40 (MBW) complex. Plant Signaling Behav. 9 (1), e27522. doi: 10.4161/psb.27522 PMC4091223 24393776

[B20] Li B. Dewey C. N. (2011). RSEM: accurate transcript quantification from RNA-Seq data with or without a reference genome. BMC Bioinf. 12, 323. doi: 10.1186/1471-2105-12-323 PMC3163565 21816040

[B21] Lightbourn G. J. Griesbach R. J. Novotny J. A. Clevidence B. A. Rao D. D. Stommel J. R. (2008). Effects of anthocyanin and carotenoid combinations on foliage and immature fruit color of *Capsicum annuum* L. J. heredity 99 (2), 105–111. doi: 10.1093/jhered/esm108 18222931

[B22] Liu J. Ai X. Wang Y. Lu Q. Li T. Wu L.. (2020). Fine mapping of the Ca3GT gene controlling anthocyanin biosynthesis in mature unripe fruit of *Capsicum annuum* L. Theor. Appl. Genet. 133 (9), 2729–2742. doi: 10.1007/s00122-020-03628-7 32564095

[B23] Liu Y. Schiff M. Dinesh-Kumar S. P. (2002). Virus-induced gene silencing in tomato. Plant J. 31 (6), 777–786. doi: 10.1046/j.1365-313x.2002.01394.x 12220268

[B24] Liu Y. Tikunov Y. Schouten R. E. Marcelis L. F. M. Visser R. G. F. Bovy A. (2018). Anthocyanin biosynthesis and degradation mechanisms in solanaceous vegetables: A review. Front. Chem. 6. doi: 10.3389/fchem.2018.00052 PMC5855062 29594099

[B25] Maloney G. S. DiNapoli K. T. Muday G. K. (2014). The anthocyanin reduced tomato mutant demonstrates the role of flavonols in tomato lateral root and root hair development. Plant Physiol. 166 (2), 614–631. doi: 10.1104/pp.114.240507 25006027 PMC4213093

[B26] Mori S. Kobayashi H. Hoshi Y. Kondo M. Nakano M. (2004). Heterologous expression of the flavonoid 3',5'-hydroxylase gene of Vinca major alters flower color in transgenic Petunia hybrida. Plant Cell Rep. 22 (6), 415–421. doi: 10.1007/s00299-003-0709-3 14504908

[B27] Panthee D. R. Gardner R. G. (2013). ‘Mountain Vineyard’hybrid grape tomato and its parents: NC 4 Grape and NC 5 Grape tomato breeding lines. HortScience 48 (9), 1189–1191. doi: 10.21273/HORTSCI.48.9.1189

[B28] Peng Y. Lin-Wang K. Cooney J. M. Wang T. Espley R. V. Allan A. C. (2019). Differential regulation of the anthocyanin profile in purple kiwifruit (Actinidia species). Horticult. Res. 6, 3. doi: 10.1038/s41438-018-0076-4 PMC6312553 30622721

[B29] Petroni K. Tonelli C. (2011). Recent advances on the regulation of anthocyanin synthesis in reproductive organs. Plant Sci. 181 (3), 219–229. doi: 10.1016/j.plantsci.2011.05.009 21763532

[B30] Qiu Z. Wang X. Gao J. Guo Y. Huang Z. Du Y. (2016). The tomato hoffman's anthocyaninless gene encodes a bHLH transcription factor involved in anthocyanin biosynthesis that is developmentally regulated and induced by low temperatures. PloS One 11 (3), e0151067. doi: 10.1371/journal.pone.0151067 26943362 PMC4778906

[B31] Sato M. Kawabe T. Hosokawa M. Tatsuzawa F. Doi M. (2011). Tissue culture-induced flower-color changes in Saintpaulia caused by excision of the transposon inserted in the flavonoid 3', 5' hydroxylase (F3'5'H) promoter. Plant Cell Rep. 30 (5), 929–939. doi: 10.1007/s00299-011-1016-z 21293860

[B32] Saxena K. B. Vales M. I. Kumar R. V. Sultana R. Srivastava R. K. (2011). Ensuring genetic purity of pigeonpea hybrids by incorporating a naked-eye polymorphic marker in A and B lines. Crop Sci. 51 (4), 1564–1570. doi: 10.2135/cropsci2010.11.0655

[B33] Stommel J. R. Lightbourn G. J. Winkel B. S. Griesbach R. J. (2009). Transcription factor families regulate the anthocyanin biosynthetic pathway in *Capsicum annuum* . J. Am. Soc. Hortic. Sci. 134 (2), 244–251. doi: 10.21273/JASHS.134.2.244

[B34] Strack D. Vogt T. Schliemann W. (2003). Recent advances in betalain research. Phytochemistry 62 (3), 247–269. doi: 10.1016/s0031-9422(02)00564-2 12620337

[B35] Su N. Hu M. L. Wu D. X. Wu F. Q. Fei G. L. Lan Y.. (2012). Disruption of a rice pentatricopeptide repeat protein causes a seedling-specific albino phenotype and its utilization to enhance seed purity in hybrid rice production. Plant Physiol. 159 (1), 227–238. doi: 10.1104/pp.112.195081 22430843 PMC3366715

[B36] Sun X. Zhang Z. Li J. Zhang H. Peng Y. Li Z. (2022). Uncovering hierarchical regulation among MYB-bHLH-WD40 proteins and manipulating anthocyanin pigmentation in rice. Int. J. Mol. Sci. 23 (15), 8203. doi: 10.3390/ijms23158203 35897779 PMC9332703

[B37] Tanaka Y. Sasaki N. Ohmiya A. (2008). Biosynthesis of plant pigments: anthocyanins, betalains and carotenoids. Plant J. 54 (4), 733–749. doi: 10.1111/j.1365-313X.2008.03447.x 18476875

[B38] Trapnell C. Williams B. A. Pertea G. Mortazavi A. Kwan G. van Baren M. J.. (2010). Transcript assembly and quantification by RNA-Seq reveals unannotated transcripts and isoform switching during cell differentiation. Nat. Biotechnol. 28 (5), 511–515. doi: 10.1038/nbt.1621 20436464 PMC3146043

[B39] Wan C. Li C. Ma X. Wang Y. Sun C. Huang R.. (2015). GRY79 encoding a putative metallo-β-lactamase-trihelix chimera is involved in chloroplast development at early seedling stage of rice. Plant Cell Rep. 34 (8), 1353–1363. doi: 10.1007/s00299-015-1792-y 25903544

[B40] Wan H. Yuan W. Ruan M. Ye Q. Wang R. Li Z.. (2011). Identification of reference genes for reverse transcription quantitative real-time PCR normalization in pepper (*Capsicum annuum* L.). Biochem. Biophys. Res. Commun. 416 (1-2), 24–30. doi: 10.1016/j.bbrc.2011.10.105 22086175

[B41] Wang D. Bosland P. W. (2006). The genes of *capsicum* . HortSci 41 (5), 1169–1187. doi: 10.21273/HORTSCI.41.5.1169

[B42] Wang Y. S. Xu Y. J. Gao L. P. Yu O. Wang X. Z. He X. J.. (2014). Functional analysis of flavonoid 3',5'-hydroxylase from tea plant (Camellia sinensis): critical role in the accumulation of catechins. BMC Plant Biol. 14, 347. doi: 10.1186/s12870-014-0347-7 25490984 PMC4275960

[B43] Wei C. Wang H. Heng S. Wen J. Yi B. Ma C.. (2019). Construction of restorer lines and molecular mapping for restorer gene of hau cytoplasmic male sterility in Brassica napus. Theor. Appl. Genet. 132 (9), 2525–2539. doi: 10.1007/s00122-019-03368-3 31165223

[B44] Zhang L. Huang Z. Wang X. Gao J. Guo Y. Du Y.. (2016a). Fine mapping and molecular marker development of anthocyanin absent, a seedling morphological marker for the selection of male sterile 10 in tomato. Mol. Breed. 36 (8), 1–10. doi: 10.1007/s11032-016-0531-6

[B45] Zhang X. Sun H. Xu Y. Chen B. Yu S. Geng S.. (2016b). Development of a large number of SSR and InDel markers and construction of a high-density genetic map based on a RIL population of Pepper (*Capsicum annuum* L.). Mol. Breed. 36 (7), 1–10. doi: 10.1007/s11032-016-0517-4

[B46] Zhao C. L. Chen Z. J. Bai X. S. Ding C. Long T. J. Wei F. G.. (2014). Structure-activity relationships of anthocyanidin glycosylation. Mol. Diversity 18 (3), 687–700. doi: 10.1007/s11030-014-9520-z 24792223

[B47] Zhao X. Zhang Y. Long T. Wang S. Yang J. (2022). Regulation mechanism of plant pigments biosynthesis: anthocyanins, carotenoids, and betalains. Metabolites 12 (9), 871. doi: 10.3390/metabo12090871 36144275 PMC9506007

