# Supplementary material for: Fine mapping of the flavonoid 3’,5’-hydroxylase gene controlling anthocyanin biosynthesis in pepper anthers and stems
Source: Front Plant Sci. 2023 Jul 27;14:1232755. doi: 10.3389/fpls.2023.1232755 (PMC10416102; doi:10.3389/fpls.2023.1232755)
Supplement: Supplementary file 1 [file Table_1.docx]

Table S1 Primers used for the qRT-PCR validation.

| Name | Forward Primer | Reverse Primer |
| --- | --- | --- |
| C4H | GATTCCTTCCATTCGGTGTT | CCTTTCTCCGTGGTGTCG |
| 4CL | CTGGACCAGTGCTGGCAAT | GGTTACGGGGCAAAGAACAA |
| CHS | GTGGAACCGTTATCCGACTAGCAA | GTATCACTTGGGCCACGGAAAGTA |
| CHI | CCTCCTGGTTCTACACCACC | CTTTGCGGCAGGTGAAACTC |
| F3'H | GGCATGTGTGGATATGGACC | CCTCCGGTGCTGGATTCTG |
| F3'5'H | TTTGAGTTGATTCCGTTTGGTGC | CTTCGAGAGGGACAGCCTTTTGC |
| DFR | AATCGCTCCAGCTGGTCTCATCAT | CTAACACAGGGAAGAGGCTGGTTT |
| ANS | CAAATGCCCACAACCAGAACTAGC | CGCACTTTGCAGTTACCCACTTTC |
| UFGT | GGATGGTGTCAAACAAGGC | GTTCAGTACAACACCATCTGC |
| *UBI-3* | CACATGTACAAGACTGACAGGGCCA | AGACCCGTTCCTTGACAACCCAC |
