# Supplementary material for: Fine mapping of the flavonoid 3’,5’-hydroxylase gene controlling anthocyanin biosynthesis in pepper anthers and stems
Source: Front Plant Sci. 2023 Jul 27;14:1232755. doi: 10.3389/fpls.2023.1232755 (PMC10416102; doi:10.3389/fpls.2023.1232755)
Supplement: Supplementary file 2 [file Table_2.docx]

Table S2 Marker types, positions and primer sequences regarding SSR and InDel markers used for mapping the *ayw* locus in the present study.

| Type | Markers names | Position in Chr11 | Forward primer | Reverse primer |
| --- | --- | --- | --- | --- |
| SSR | genSSR5929 | 252,860,899-252,869,177 | ATCTTAGCAAGAATGCCCCA | CGGTAACCCATGTCACGATT |
| SSR | genSSR5955 | 254,147,690-254,149,121 | GGGAGTGAAGAATGAACGGA | TACACGCAAAGCCTTCTTCC |
| InDel | CIDH351 | 251,906,806-251,907,010 | ACGTGCAGTAATCAGCGTGA | GTGAAACGACGGATCTGGAGA |
| SSR | PS146 | 252,560,110-252,560,319 | AGAAAGGCCTCCAAACAACC | CGCCGAGCACTACTCTATCC |
| SSR | ZS161629 | 251,908,626-251,908,640 | CAAGCTTTCATCGAAGACAAAA | AGGTTTCAACCACTCCCAAA |
| SSR | ZS161713 | 252,694,408-252,694,452 | AACCTTGTGCAATAAAAGCG | AACCCCCATTCCTATCAACC |
| SSR | ZS161721 | 252,728,127-252,728,146 | CATTTTCCCCACCTTTTCAA | CGAACAAATTGAGGGGTGAC |
| SSR | ZS161820 | 253,531,009-253,531,026 | TCCACACATCCGAAGGGTAT | CGCAAAGCTCCTCAAACTTC |
| SSR | GSSR70 | 252,863,689-252,887,009 | TGATGTGCATTTCCTGTGGT | TAGACTCCGTTTTTCGCTGC |
