# Supplementary material for: Fine mapping of the flavonoid 3’,5’-hydroxylase gene controlling anthocyanin biosynthesis in pepper anthers and stems
Source: Front Plant Sci. 2023 Jul 27;14:1232755. doi: 10.3389/fpls.2023.1232755 (PMC10416102; doi:10.3389/fpls.2023.1232755)
Supplement: Supplementary file 3 [file Table_3.docx]

Table S3 Positions and primer sequences regarding KASP markers used for mapping the ayw locus in the present study.

| Names | Position in Chr11 | Primer_AlleleX | Primer_AlleleY | Primer_Common |
| --- | --- | --- | --- | --- |
| aywSNP-120 | 254593097 | GAAGGTGACCAAGTTCATGCTCCCAAGTGAAGTAGAGAGGCTC | GAAGGTCGGAGTCAACGGATTCCCAAGTGAAGTAGAGAGGCTG | ATACTGTTATCGGTCCTAAACCGGG |
| aywSNP550 | 254753129 | GAAGGTGACCAAGTTCATGCTTTTTCCAATAATTTGGTCCGTTTCTTG | GAAGGTCGGAGTCAACGGATTTTTCCAATAATTTGGTCCGTTTCTTC | GGCACTTGCAGAAATGATGAAAAATTCAAC |
| aywSNP-124 | 254791794 | GAAGGTGACCAAGTTCATGCTAAAATTATACTATATATATAAGATCACCTTTATCTA | GAAGGTCGGAGTCAACGGATTAATTATACTATATATATAAGATCACCTTTATCTG | TTTCTAAACCTTAAAAATAAGACATCCTATTTGC |
| aywSNP-99 | 254877722 | GAAGGTGACCAAGTTCATGCTCGTAAAGAAAGAAGAATACCCAGCAAT | GAAGGTCGGAGTCAACGGATTGTAAAGAAAGAAGAATACCCAGCAAG | GATTTTCCCGTTTATGGCCGTTCTG |
